# Do ectomycorrhizal exploration types reflect mycelial foraging strategies?

**DOI:** 10.1111/nph.18566

**Published:** 2022-11-23

**Authors:** Karolina Jörgensen, Karina E. Clemmensen, Håkan Wallander, Björn D. Lindahl

**Affiliations:** ^1^ Department of Soil and Environment Swedish University of Agricultural Sciences Box 7014 SE‐750 07 Uppsala Sweden; ^2^ Department of Biological Sciences University of Bergen Box 7803 NO‐5020 Bergen Norway; ^3^ Department of Forest Mycology and Plant Pathology Swedish University of Agricultural Sciences Box 7026 SE‐750 07 Uppsala Sweden; ^4^ Department of Biology Lund University Sölvegatan 37 223 26 Lund Sweden

**Keywords:** boreal forest, cafeteria experiment, ectomycorrhizal exploration types, fungal networks, nutrient foraging, soil fungi

## Abstract

- Ectomycorrhizal exploration types are commonly assumed to denote spatial foraging patterns and resource‐related niches of extraradical mycelia. However, empirical evidence of the consistency of foraging strategies within exploration types is lacking.
- Here, we analysed ectomycorrhizal foraging patterns by incubating root‐excluding ingrowth mesh bags filled with six different substrates in mature *Picea abies* forests. High‐throughput sequencing was used to characterise ectomycorrhizal fungal communities in the mesh bags and on adjacent fine roots after one growing season.
- Contrary to expectations, many ectomycorrhizal genera of exploration types that are thought to produce little extraradical mycelium colonised ingrowth bags extensively, whereas genera commonly associated with ample mycelial production occurred sparsely in ingrowth bags relative to their abundance on roots.
- Previous assumptions about soil foraging patterns of exploration types do not seem to hold. Instead, we propose that variation in the proliferation of extraradical mycelium is related to intergeneric differences in mycelial longevity and the mobility of targeted resources.

## Introduction

Ectomycorrhizal fungi link tree roots to the soil environment by forming extraradical mycelium that extends into the soil from the mycorrhizal root tip (Smith & Read, 2008). The extent and morphology of the extraradical mycelium are important traits that may be linked to functional variation among species and genera (Agerer, 2001), which may relate further to ecosystem processes. For example, mycelium is an important precursor of soil organic matter (Clemmensen *et al*., 2013; Adamczyk *et al*., 2019), and morphological differences have been linked to decomposer capacity (Clemmensen *et al*., 2021; Argiroff *et al*., 2022). Ectomycorrhizal fungi can be classified into different ‘soil exploration types’ based on general morphological traits of the colonised root tips and emanating mycelium. These exploration types have been hypothesised to reflect the extent and manner of extraradical mycelial proliferation in the soil. The ‘contact’ type is described as having dense, smooth, hydrophilic mantles and only few emanating hyphae, while the ‘short‐distance’ type produces abundant short, nonaggregated hyphae in the near vicinity of the root tip. By contrast, the ‘medium‐distance smooth’ and ‘long‐distance’ types produce little extraradical mycelium close to the root but form cords, which vary in length and hydrophobicity. The ‘medium‐distance fringe’ and ‘mat’ types form extensive mycelia with many aggregated, hydrophobic cords (Agerer, 2001). The medium‐distance fringe, mat and long‐distance types have been associated with high mycelial biomass production (Hobbie & Agerer, 2010).

Exploration types have, to some extent, been found to reflect niche differentiation of ectomycorrhizal fungi. Those with none or few cords (contact, short‐distance and medium‐distance smooth) have been proposed to maximise the area of hydrophilic hyphae that extend into the soil and thereby promote rapid uptake of mobile N and decrease leaching (Hobbie & Agerer, 2010; Bahr *et al*., 2015). Types with hydrophobic mantles and cords (medium‐distance fringe and long‐distance) may instead display more directed growth towards discrete patches of immobile, organic resources (Finlay & Read, 1986; Cairney, 2005; Hobbie & Agerer, 2010). Furthermore, ectomycorrhizal fungi may respond to small‐scale variation in substrate quality by adapting local mycelial proliferation and spatial distribution (Rosling *et al*., 2004; Kluting *et al*., 2019). Exploration types have also been suggested to reflect patterns of community assembly via their different abilities to colonise new roots, with cord‐forming types being more successful in habitats with low root density (Peay *et al*., 2011).

It is important to point out that the exploration types originally were defined based on morphological investigations of root tips (Agerer, 2001) and hypotheses about the extent and foraging patterns of extraradical mycelia have largely been extrapolated from the amount and morphology of hyphae emanating from the mantle and often based on a few species (Weigt *et al*., 2011). Although exploration types are used as equivalents to foraging strategies (Tedersoo & Smith, 2013), whether they correspond to systematic and consistent differences in soil foraging remains uncertain. In comparisons between fungal communities on roots and in soil, species forming contact‐type mycorrhizas were underrepresented in the soil (Genney *et al*., 2006; Kjøller, 2006), implying a poor ability to forage for nutrients away from the roots. Kjøller (2006) found that species with exploration types assumed to form extraradical mycelia (short‐distance, medium‐distance and long‐distance) occurred abundantly in root‐free ingrowth mesh bags relative to roots. Similarly, Parrent & Vilgalys (2007) found that *Tylospora* (short‐distance) and *Amanita* (medium‐distance smooth) were prolific in ingrowth mesh bags and in bulk soil, although rarely observed as ectomycorrhizas, suggesting a high capacity to forage for nutrients away from the roots. Despite similarities in the results of these studies, divergent patterns have been observed for the long‐distance genus *Suillus*. Parrent & Vilgalys (2007) found *Suillus* to be an extensive soil coloniser despite being rare on roots, while Genney *et al*. (2006) observed the opposite relationship. Thus, more empirical evidence is needed to underpin a better understanding of how exploration types relate to proliferation of extraradical mycelium and selective colonisation of soil niches.

In this study, we conducted a ‘cafeteria experiment’ (Krebs, 1999), in which we incubated root‐excluding mycelial ingrowth mesh bags filled with different soil and sand substrates in mature, boreal *Picea abies* forests during one growing season. We investigated whether ectomycorrhizal fungi assigned to the same exploration type share common foraging patterns by comparing ectomycorrhizal communities in the ingrowth bags with those on adjacent fine roots, using DNA‐based metabarcoding. DNA has many drawbacks as a marker of mycelial biomass (Baldrian *et al*., 2013). For instance, copy numbers per hyphal material vary between taxa and most likely also between different tissue types within taxa. Nevertheless, DNA metabarcoding enables semiquantitative assessment (Castaño *et al*., 2020) of colonisation patterns of different fungal taxa and is particularly useful to evaluate relative differences among communities. Furthermore, we can provide information for taxa that are difficult to isolate, for which information from laboratory microcosms is scarce. The cafeteria experiment setup has been used commonly in animal ecology, where inference of ecology can be made based on the choices of animals when offered different food sources. Here, we used a similar methodology to investigate the extent and selectivity of extraradical mycelial foraging by different ectomycorrhizal fungi. The cafeterias offered a set of six different mesh bags, filled either with soil with varying pH and organic matter and nutrient contents, or with inert sand with or without apatite as a phosphorus (P) source. To capture a larger diversity of fungal species and foraging traits across different environments, cafeterias were incubated in 10 forests that varied in soil pH and inorganic N availability.

Based on the general morphological traits of their exploration types, we hypothesised that ectomycorrhizal fungi forage for soil resources in different ways. Contact type mycorrhizal fungi were expected to have low abundance in all bags due to their limited extension into the soil. The short‐distance and medium‐distance smooth exploration types, which form hydrophilic mycelia that absorb soluble, low‐molecular‐size nutrients from the surrounding soil, were expected to colonise all substrates (including inert sand) without preference, in a space‐filling manner. The hydrophobic medium‐distance fringe and long‐distance exploration types are thought to forage for immobile organic resources by forming cords and are expected to explore the ingrowth bags, particularly those with soil, with extensively proliferating extraradical mycelium (Finlay & Read, 1986; Leake *et al*., 2001) in a manner analogous to saprotrophic cord‐forming fungi (Boddy, 1999).

Furthermore, if fungal micro‐niches are determined by direct and local environmental filtering of mycelial colonisation (Rosling *et al*., 2004; Kluting *et al*., 2019), we expected fungal taxa to diverge in their colonisation of different soil substrates. Specifically, we expected species of short‐distance and medium‐distance smooth types, which are relatively more abundant in nutrient‐rich environments (Moeller *et al*., 2014; Sterkenburg *et al*., 2015; Defrenne *et al*., 2019; Pellitier & Zak, 2021), to predominantly colonise more nutrient‐rich soil substrates, also on the local ‘cafeteria scale’. By contrast, we expected cord‐forming species (mainly of medium‐fringe type), which are often abundant in nutrient‐poor environments, to be relatively more abundant in nutrient‐poor substrates due to lower competition from other ectomycorrhizal fungi.

Finally, we amended sand bags with apatite to investigate preferential colonisation by specific genera potentially involved in P mining by mineral weathering. Such a trait may be a competitive advantage in N‐rich but P‐limited forests, and increased ectomycorrhizal biomass production has been observed in apatite‐amended ingrowth bags in nemo‐boreal *P. abies* forests subjected to high rates of N deposition (14.5 kg ha^−1^ yr^−1^) (Wallander & Thelin, 2008; Almeida *et al*., 2019).

## Materials and Methods

### Ingrowth mesh bags

Aiming for a high variation in soil properties, organic topsoil or mull‐rich mineral soil (top 10 cm) was collected in autumn 2017 from four Swedish *Picea abies* (L.) H. Karst forests. Two soils were collected in central Sweden and two in southern Sweden. One of the southern soils had been subjected to P fertilisation. Some chemical characteristics of the soils are described in more detail in Table 1. Green parts of mosses and roots coarser than 2 mm in diameter were removed, and the soils were stored in bags at room temperature and in darkness for 17 months to reduce background levels of ectomycorrhizal DNA (Bååth *et al*., 2004). The soils remained moist throughout the storage period.

**Table 1:** Characteristics of substrates used in the ingrowth mesh bags in the cafeteria experiment.

Substrate	N deposition (kg ha^−1^ yr^−1^)	At collection	After 17 months preincubation				
		pH	pH	Mineralised N (μg g^−1^ OM)	OM (%)	C/N	EMF DNA (%)
Organic, central Sweden	5	4.2	4.4	2540	63	26	5.6
Mull soil, central Sweden	5	6.1	4.9	3220	8	16	4.5
Organic, southern Sweden	10–15	4.1	4.9	2090	66	25	0.6
P‐fertilised organic, southern Sweden	10–15	3.6	4.9	1340	71	29	3.1
Sand	–	–	–	–	0	–	0
Sand with 1% apatite	–	–	–	–	0	–	0

The soil was collected from mature *Picea abies* forests in two areas with contrasting atmospheric N deposition levels. pH was measured at the time of collection and after 17 months of preincubation. Mineralised N was calculated as the difference in inorganic N between un‐sieved, wet soils and sieved, dried soils after preincubation. Organic matter (OM) % and C/N were measured after preincubation. The proportion of ectomycorrhizal fungal (EMF) DNA was acquired through sequencing of ITS2 markers.

After 17 months, the soils were frozen, ground in a custom‐built freeze‐mill, sieved through 2 mm mesh, soaked in deionised water to wash out soluble nutrients and drained. The soils were dried at 40°C and filled into cylindrical mesh bags (8 cm long, 2.5 cm diameter; 50 μm mesh size; Sintab Product AB, Malmö, Sweden), which allowed ingrowth of fungal mycelium but excluded tree roots (Wallander *et al*., 2001); *c*. 7 g of organic topsoil and *c*. 21 g of mull soil were used to fill the bags. Additional bags were filled with either sand (*c*. 40 g; 0.36–2.0 mm; 99.6% SiO_2_; Silversand 90; Sibelco Nordic AB, Västerås, Sweden) or sand mixed with 1% apatite (Krantz, Bonn, Germany; sourced from Madagascar) with a grain size of 0.65–2.00 mm.

pH of the soil substrates was measured in a 1 : 5 v/v ratio of soil and deionised H_2_O with an 855 Robotic Titrosampler and an Aquatrode Plus combined pH electrode (Metrohm, Herisau, Switzerland) at collection and after 17 months of preincubation in room temperature. After sieving, washing and drying the preincubated soils, inorganic N concentrations (NH_4_^+^ and NO_3_^−^) were measured by extraction in a 1 : 2.5 w/w ratio of soil and 2 M KCl and analysed on an autoanalyser (Bran + Luebbe XY‐2 Sampler; Seal Analytical Inc., Emu Plains, NSW, Australia). Organic matter concentration was determined by loss on ignition at 550°C for 5 h, and C/N was measured in a combustion elemental analyser (TruMac CN; Leco, St Joseph, MI, USA).

### Site selection and field incubation

Ten mature (> 70 yr) *P. abies‐*dominated forests in central Sweden (latitude: 59.2–60.5°N) were selected for field incubation (Table 2). Forests with contrasting soil fertility were selected based on visual assessments (e.g. composition of understorey vegetation, contribution of deciduous trees and soil type). More productive sites had moder/mineral soils, some contribution of deciduous trees (*Betula*, *Populus*, *Corylus*) and an understorey consisting of mosses (*Rhytidiadelphus* sp., *Hylocomium splendens*, *Ptilium crista‐castrensis* and *Pleurozium schreberi*), grasses and ferns. Less productive sites had podzolised soil with a distinct organic layer (organic topsoil) overlying mineral soil, some contribution of *Pinus sylvestris* and understorey vegetation consisting of mosses (*Hylocomium splendens* and *Pleurozium schreberi*) and dwarf shrubs (*Vaccinium myrtillus* and *Vaccinium vitis*‐*idaea*).

**Table 2:** Characteristics of the forests used for the incubation of the cafeterias.

Soil type	Coordinates (WSG 84)		Basal area (m^2^ ha^−1^)	NH_4_^+^–N (μg g^−1^ OM)	NO_3_^−^–N (μg g^−1^ OM)	pH
	Latitude	Longitude				
Podzol	59.27	14.84	42.9	24.8 (26.1)	0.5 (0.4)	4.1 (0.5)
Podzol	59.69	14.88	39.3	24.9 (19.2)	0.4 (0.3)	4.2 (0.2)
Podzol	60.29	17.05	36.0	19.2 (4.5)	0.5 (0.2)	4.4 (0.3)
Podzol	60.28	17.74	34.0	18.4 (20.5)	0.2 (0.1)	4.5 (0.2)
Mull	59.62	15.20	53.5	18.6 (9.4)	0.1 (0.1)	4.8 (0.2)
Podzol	60.07	17.80	44.1	29.6 (9.6)	0.3 (0.2)	5.1 (0.8)
Mull	59.88	17.35	N/A	23.8 (14.7)	1.1 (2.5)	5.2 (0.3)
Mull	59.96	18.19	37.9	34.1 (8)	0.1 (0.2)	5.5 (0.3)
Mull	60.18	17.86	28.3	20.6 (10.8)	0 (0)	5.6 (0.3)
Mull	60.55	17.95	35.7	63.7 (21.1)	0.5 (0.2)	6.4 (0.8)

Basal area was determined by measuring the diameter at breast height of all living trees within a 10 m radius from the central point of the plots. Soil characteristics were determined from one soil core taken in the centre of each cafeteria representing the same depth as the bags. The numbers in parentheses are within‐stand standard deviations of *n* = 5 cafeterias.

Bags were soaked in deionised H_2_O for a couple of minutes and placed in holes made by removing a soil core (2.5 cm in diameter) with a metal soil corer. At sites without a distinct organic layer, bags were placed vertically down to 8 cm depth from the soil surface and at sites with podzol soils, they were placed with the bottom of the bag at the organic–mineral soil interface. The six bags, containing different substrates (Table 1), were grouped in five replicate ‘cafeterias’ per site, spaced at least 5 m apart, each containing one bag of each substrate in a circle with 1 m diameter and even spacing of bags (Supporting Information Fig. S1). In total, 10 sites × 6 substrates × 5 replicates = 300 bags were incubated.

The incubation period lasted 153–160 d (May–November), after which the bags were retrieved from the soil (277 bags were recovered), placed individually in 50 ml tubes and frozen at −20°C within 8 h. At the time of bag collection, two soil cores (3 cm in diameter) were sampled from the middle of each cafeteria, spanning the same depth as the bags.

### Sample preparation and soil chemical analysis

One of the two soil cores per cafeteria was used to measure soil chemical characteristics. The core was gently homogenised before a subsample (5 ml) was used to analyse pH as described previously. Another subsample was used to extract ammonium and nitrate as described previously. *Picea abies* roots (< 2 mm in diameter) were retrieved from the second central soil core and rinsed carefully. Cleaned roots and substrates from ingrowth mesh bags were freeze‐dried and finely ground; soils and roots in a Precellys homogenizer (Bertin Instruments, Montigny la Bretonneux, France); and sand in a ball mill (LMLW‐320/2; Laarmann, Roermond, the Netherlands).

### Fungal community analysis

DNA was extracted from 20 to 50 mg (roots), 75 mg (organic soils: central, southern, southern P fertilised), 250 mg (mull soil) or 500 mg (sand and sand amended with apatite) of material with the NucleoSpin Soil kit (Macherey‐Nagel, Duren, Germany), and extracts were diluted to a DNA concentration of 0.5 ng μl^−1^. DNA was also extracted from samples of substrates that were not incubated in the field. Amplicons of the ITS2 region were produced by PCR using the forward primer fITS7 (Ihrmark *et al*., 2012) and the reverse primer ITS4 (White *et al*., 1990) with unique identification tags attached to both primers. Reactions (50 μl) were run with 12.5 ng of DNA template, 0.2 mM dNTP, 0.025 U μl^−1^ Dreamtaq polymerase (Thermo Fischer Scientific, Waltham, MA, USA) and 0.5 μM of each primer. PCR was performed with denaturation at 94°C, annealing at 56°C and extension at 72°C for 30 s each and cycle numbers (28–35) optimised to ensure that the reaction was in the exponential phase (Castaño *et al*., 2020). Negative controls with deionised H_2_O instead of DNA template were included. A total of 267 ingrowth mesh bag samples, 46 root samples and samples of nonincubated substrates (to characterise background communities) were successfully amplified and cleaned with Sera‐Mag (Cytiva, Marlborough, MA, USA) according to the manufacturer's instructions. Amplicon concentrations were measured fluorometrically on a Qubit (Invitrogen), and the PCR products were merged in equal amounts into four pools and cleaned with the EZNA Cycle Pure Kit (Omega Bio‐Tek., Norcross, GA, USA). Library preparation and sequencing were conducted by SciLifeLab (NGI, Uppsala, Sweden) on the PacBio Sequel I platform (Pacific Biosciences, Menlo Park, CA, USA) in one SMRT cell per pool. The PacBio platform was chosen to minimise biases due to ITS2 length variation (Castaño *et al*., 2020). Raw sequences were filtered and clustered in the bioinformatics pipeline Scata (https://scata.mykopat.slu.se/; Ihrmark *et al*., 2012), accepting sequences with length > 100 bp, mean quality > 20, single base quality > 3, primer sequence similarity > 90% and intact identification tags. Genotypes found only once in the whole data set were removed, and sequences were clustered into species hypotheses (Kõljalg *et al*., 2013) using single‐linkage clustering with 98.5% similarity to the closest neighbour required for sequences to join clusters.

Species were identified by comparing representative sequences to the UNITE database (Nilsson *et al*., 2019), and only ectomycorrhizal fungal species hypotheses (*n* = 342) were selected for further analyses. Relative abundances of any ectomycorrhizal species (in the total fungal community) found in soil substrates before field incubations (i.e. background levels, 0.6–5.6%; Table 1) were subtracted from their relative abundances measured after incubation (31–43%). After this background correction, relative abundances of ectomycorrhizal genera were calculated as their share of the ectomycorrhizal community. We selected 12 genera that were present on roots in at least 10 cafeterias (out of a total of 50) to be included in the statistical analyses. Exploration types were assigned according to Tedersoo & Smith (2013) and the DEEMY database (Agerer & Rambold, 2004; http://www.deemy.de/), and in cases of known intrageneric variation, the exploration type of the dominant species was used. The tested genera and their assigned exploration types, hydrophobicity and expected foraging patterns are listed in Table 3.

**Table 3:** Functional traits of the 12 most frequent ectomycorrhizal genera.

Genus	Exploration type	Hydrophobicity	Expectation
*Amanita*	Medium‐distance smooth	Hydrophobic	Uncertain
*Amphinema*	Medium‐distance fringe	Hydrophobic	Prolific in soil bags
*Cenococcum*	Short‐distance	Hydrophilic	Prolific in all bags
*Cortinarius*	Medium‐distance fringe	Hydrophobic	Prolific in soil bags
*Hyaloscypha*	Contact	Hydrophilic	Little colonisation of bags
*Hygrophorus*	Short‐distance		Prolific in all bags
*Lactarius*	Contact	Hydrophilic	Little colonisation of bags
*Piloderma*	Medium‐distance fringe	Hydrophobic	Prolific in soil bags
*Pseudotomentella*	Medium‐distance smooth	Hydrophilic	Prolific in all bags
*Russula*	Contact	Hydrophilic	Little colonisation of bags
*Tomentella*	Medium‐distance smooth	Hydrophilic	Prolific in all bags
*Tylospora*	Short‐distance	Hydrophilic	Prolific in all bags

Exploration types, hydrophobicity (based on Lilleskov *et al*., 2011) and expected extraradical mycelial proliferation of the 12 most frequent genera in the cafeteria study.

### Statistical analysis

For each ectomycorrhizal genus in each cafeteria, we calculated a log ratio of the relative abundance in ingrowth bags relative to roots (Eqn 1) and in soil bags relative to sand bags (Eqn 2). Only cafeterias where the genus was present on roots were included in these calculations, with 11–39 cafeterias assessed depending on the genus.

(Eqn 1) $$\log\left(\frac{\text{mean abundance}\;\mathrm{all}\;\text{bags}+\mu }{\text{abundance}\;\mathrm{on}\;\text{roots}}\right)$$

(Eqn 2) $$\;\log\left(\frac{\text{mean abundance soil bags}+\mu }{\text{mean abundance sand bags}+\mu }\right)$$

where μ = 1/(mean sequencing depth × 6), that is the lowest expected relative abundance, was added to avoid zeros.

To evaluate whether exploration type was a good predictor for mycelial growth patterns, we used mixed‐effect linear models (the lmertest and lme4 packages) (Bates *et al*., 2015; Kuznetsova *et al*., 2017) in R (v.4.0.3; R Core Team, 2020) with the log ratios as response variables, exploration type as explanatory variable and genus, and cafeteria nested within site as random factors. Next, the log ratios for each genus were tested to investigate whether individual genera were characterised by little or prolific extraradical mycelial growth (i.e. low or high soil/root ratio) or a preference for soil over inert sand. This was done by testing whether the intercept of mixed‐effect linear models with log ratio as response variable and cafeteria nested within site as random factor was significantly different from zero. *P*‐values from all genera‐specific models were corrected for testing of multiple taxa by the Benjamini–Hochberg method (Benjamini & Hochberg, 1995). We also tested whether different types of substrates recruited different ectomycorrhizal communities, that is if the extraradical mycelia of different genera displayed preference for specific substrates. Substrate effects on community composition at the genus level among soil bags (humus and mull substrates) and among sand bags (sand and apatite amended sand) were evaluated by PERMANOVA (adonis2 function in the vegan package in R; Oksanen *et al*., 2022) with 1000 permutations constrained to samples within each cafeteria. Individual mixed‐effect linear models were applied for specific genera with square root transformed relative abundance (Hellinger transformation) as the response variable, substrate as a fixed factor and cafeteria nested within site as a random factor. *P*‐values were corrected for multiple testing as described previously, and genera with *P* ≤ 0.05 were subjected to Tukey's HSD *post hoc* tests with the emmeans package (Lenth *et al*., 2021). Graphs were produced with ggplot2 (Wickham *et al*., 2020).

## Results

A total of 903 787 sequences passed the quality check, and after the removal of unique sequences, 466 080 sequences were clustered into 2668 species hypotheses, whereof 342 (272 499 sequences accounting for 0.6–95% of total reads from each sample; mean 46% and median 44%) were ectomycorrhizal and 235 (244 713 sequences accounting for 0.2–95% of total reads from each sample; mean 41% and median 38%) belonged to the 12 most frequently encountered genera (Table 3). These genera together accounted for on average 87% of the ectomycorrhizal reads in individual samples (range: 9–100%; median: 97%).

**Fig. 1:**
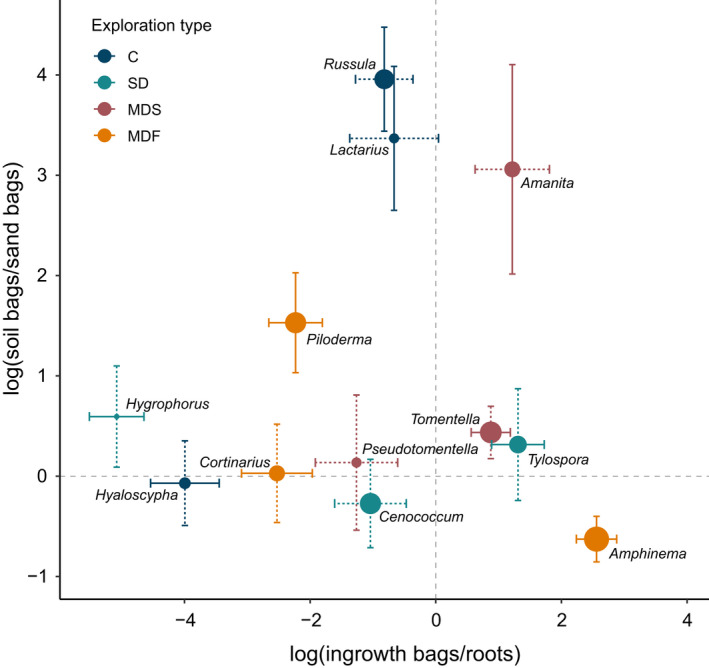
Model estimated log ratios of ectomycorrhizal genera. Log ratio was calculated as the log‐transformed ratio of the relative abundances of each genus in ingrowth bags and on roots (*x*‐axis) and in soil bags and sand bags (*y*‐axis). Point sizes correspond to the overall relative abundance of each genus in the ectomycorrhizal community. C, contact; MDF, medium‐distance fringe; MDS, medium‐distance smooth; SD, short‐distance. Dashed vertical and horizontal lines denote equal abundances in bags and on roots, and in soil bags and sand bags respectively. Error bars show SE, and solid bars denote a significant difference from zero lines (Benjamini–Hochberg‐adjusted *P* ≤ 0.05) based on mixed‐effect linear models. Model outputs are reported in Supporting Information Tables S1, S3, and S4, and relative abundances are reported in Table S2.

Exploration type was not a good predictor of relative colonisation of bags vs roots (*P* = 0.7) or soil bags vs sand bags (*P* = 0.3; Table S1). *Amphinema*, *Tomentella* and *Tylospora* had a high ratio of extraradical mycelium to root‐associated mycelium and were, thus, prolific bag colonisers, despite being of different exploration types. By contrast, *Hyaloscypha*, *Hygrophorus, Cortinarius* and *Piloderma* were more abundant on roots than as extraradical mycelium, although the two latter genera are of medium‐distance fringe type and, thus, expected to proliferate far into the soil (Tables S2, S3). *Lactarius*, *Russula, Amanita* and *Piloderma* showed a preference for soil bags, whereas *Amphinema* was more prolific in sand bags. Furthermore, *Lactarius* and *Russula* occurred as abundantly in ingrowth bags as on roots, despite being of the contact type (Fig. 1; Tables S2, S4).

**Fig. 2:**
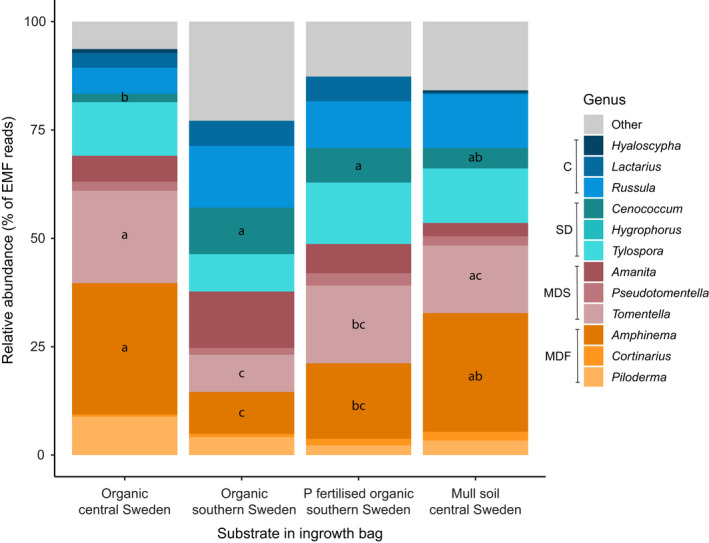
Ectomycorrhizal community composition in soil bags. Relative abundances of the 12 most frequent ectomycorrhizal genera colonising ingrowth bags filled with four different soil substrates. C, contact; MDF, medium‐distance fringe; MDS, medium‐distance smooth; SD, short‐distance. Different lowercase letters indicate significant effects (*P* ≤ 0.05) among substrate types for the respective genus according to *post hoc* Tukey HSD tests. Relative abundances are reported in Table S6, and model outputs are reported in Tables S7 and S8.

Community composition on the genus level differed between different types of soil bags (*P* = 0.001; Table S5), with three genera displaying a preference for some of the soil substrates; *Amphinema* and *Tomentella* were less abundant in the southern soils, where *Cenococcum* was more prolific (Fig. 2; Tables S6–S8). Apatite‐amended bags did not diverge in community composition from nonamended sand (*P* = 0.4; Tables S9, S10).

## Discussion

All in all, we found little support for the utility of exploration types to predict patterns of extraradical mycelial foraging. Contrary to our hypothesis, contact‐type genera did not generally colonise bags to a lesser extent than others (Fig. 1); only the ascomycete genus *Hyaloscypha* behaved as expected for a contact type and colonised roots more extensively than ingrowth bags. The other two contact‐type genera, *Russula* and *Lactarius*, preferably colonised soil substrates over sand (Fig. 1), which may explain why Kjøller (2006), who used sandbags, concluded that these genera have limited mycelial proliferation. The detection of abundant DNA of some contact‐type genera in soil substrates suggests that they (Russulaceae) may colonise the soil matrix with extraradical mycelium more extensively than proposed by Agerer (2001), in line with the observation that *Lactarius rufus* was present in bulk soil without being detected on ectomycorrhizal root tips (Genney *et al*., 2006). Presumably, contact types may extend from the roots with fine emanating hyphae that are not readily visible and selectively target organic hotspots. *Amanita* (medium‐distance smooth) behaved similarly.

We hypothesised that the extraradical mycelium of the hydrophilic short‐distance and medium‐distance smooth exploration types would expand throughout the soil without preference for any particular substrate in a space‐filling manner, and, thus, be prolific in all types of ingrowth bags. *Tomentella* (medium‐distance smooth) and *Tylospora* (short‐distance) were indeed more abundant in bags than on roots. However, the short‐distance type *Hygrophorus* was, by contrast, mainly found on roots.

Contrary to our expectations, *Piloderma* and *Cortinarius*, which both mainly form medium‐distance fringe type mycorrhizas, did not proliferate extensively in the ingrowth bags, despite being widely considered to produce large amounts of extraradical mycelial biomass (Hobbie & Agerer, 2010). On the contrary, *Amphinema* (also medium‐distance fringe) colonised bags vigorously, despite its scarce representation on root tips. Furthermore, we did not find consistent support for the hypothesis that hydrophobic, cord‐forming genera would prefer organic substrates over sand bags; *Piloderma* was, as expected, more abundant in soil bags, but *Cortinarius* had no significant preference. *Amphinema* was even preferentially found in sand bags, potentially being outcompeted (or diluted) by more selective foragers in soil bags (Fig. 1).

*Amphinema, Tomentella* and *Cenococcum* differed significantly in relative abundance between ingrowth bags with different types of soil. Although the underlying mechanism is not clear, this observation suggests that some ectomycorrhizal fungi can detect and selectively direct extraradical mycelial growth towards specific substrates, and/or that mycelial colonisation may be confined to specific micro‐niches by local environmental filtering (Rosling *et al*., 2004). However, contrary to our hypothesis, short‐distance and medium‐distance smooth types, which are supposedly nitrophilic (Moeller *et al*., 2014; Sterkenburg *et al*., 2015; Defrenne *et al*., 2019; Pellitier & Zak, 2021), did not preferably colonise the mull soil, which had the lowest C : N ratio and highest inorganic N mineralisation (Table 2). The lack of community response to apatite amendment in sand bags is concordant with the results of Hedh *et al*. (2008), possibly indicating a low demand for mineral‐bound P or large functional redundancy in terms of weathering.

Although we conclude that exploration types are not consistent predictors of soil foraging, we observed systematic differences among genera regarding their extraradical mycelial proliferation in different substrates. Tedersoo *et al*. (2012) claimed that ectomycorrhizal lineage is a better predictor of functional attributes than exploration type. However, we also observed contrasting patterns within lineages (e.g. *Piloderma* vs *Tylospora* and *Amphinema* in the Athelioid lineage).

*Piloderma* and *Cortinarius* have been highlighted as having high extraradical biomass, but we rather observed low extraradical proliferation (DNA in the bags) of these genera. Still, these genera often dominate ectomycorrhizal fungal communities and attain a high biomass in old, nutrient‐limited boreal forests (Sterkenburg *et al*., 2015; Kyaschenko *et al*., 2017). Accumulation of extraradical mycelial biomass does not depend solely on growth rate, but also on biomass turnover (Clemmensen *et al*., 2013, 2015; Ekblad *et al*., 2013; Hagenbo *et al*., 2017). Species with slow turnover of extraradical hyphae, for example by forming long‐lived cords (Treseder *et al*., 2005), may attain high biomass over a long period of time in spite of slow growth. Here, we studied colonisation of bags during only one growing season while Hagenbo *et al*. (2018) studied successional colonisation of bags over a longer period and observed that *Cortinarius* progressively increased over multiple years, suggesting slow but persistent net accumulation of perennial mycelial biomass. Furthermore, *Piloderma* selectively colonised soil substrates with organic resources, while *Cortinarius* did not display such preference. Members of *Cortinarius* are known for their capacity to decompose and derive nutrients from complex organic substrates (Bödeker *et al*., 2014; Lindahl *et al*., 2021). These two genera are also recognised as nitrophobic (Lilleskov *et al*., 2011; van der Linde *et al*., 2018), as is *Hygrophorus* (Solly *et al*., 2017). By contrast, the genera that proliferated extensively in soil bags, relative to their more scarce representation on roots, that is *Amphinema*, *Tylospora* and *Tomentella*, have often been described as nitrophilic (Kranabetter *et al*., 2009; Sterkenburg *et al*., 2015; Hagenbo *et al*., 2018), at least in a boreal context with low nitrogen deposition. *Amphinema* was also more abundant in sand bags than in soil bags, suggesting a space‐filling growth strategy. Ample production of extraradical mycelium may be advantageous at high levels of mobile, inorganic nutrients, by minimising leaching and retaining nutrients in the mycorrhizal system (Hobbie & Agerer, 2010; Bahr *et al*., 2015). However, *Amphinema* has hydrophobic cords, implying that this growth strategy is not restricted to noncord‐forming, hydrophilic exploration types.

As exploration types do not seem to be consistent predictors of mycelial foraging, we see a need for alternative frameworks, for example based on nitrophobicity and/or hydrophobicity (Almeida *et al*., 2022). Most low‐proliferating taxa in our study are recognised as nitrophobic, hydrophobic and linked to exploitation of solid organic resources in nutrient‐limited environments. A long mycelial lifespan may enable them to accumulate high biomass over time (Hagenbo *et al*., 2017) in spite of scarce resource availability. The high‐proliferating taxa, on the contrary, are relatively nitrophilic/hydrophilic and may rather employ a space‐filling strategy to minimise losses of soluble inorganic nutrients under rich conditions. These coordinated traits are likely to be continuously distributed along a trait axis (van der Linde *et al*., 2018) similar to the leaf economics spectrum of plants (Wright *et al*., 2004). Further characterisation of trait axes will facilitate understanding of ecological niches, plasticity and adaptations of ectomycorrhizal fungal species along environmental gradients. To this end, more studies are needed to assess ectomycorrhizal foraging patterns in other types of environments, ideally also with more directly quantitative methods, as relative DNA abundances are only indirectly linked to mycelial biomass.

## Author contributions

KJ, KC, HW and BL designed the study. KJ collected the data and performed the analyses. KJ wrote the first draft of the manuscript. All authors contributed to the interpretation and writing.

## Supporting information


**Fig. S1** Illustration of cafeteria setup.
**Table S1** Model output from lme‐models of the log‐ratio of ectomycorrhizal exploration types in bags relative to roots, and in soil bags relative to sand bags.
**Table S2** Relative abundance of ectomycorrhizal fungi on roots, and sand‐ or soil‐filled ingrowth bags.
**Table S3** Model output from lme‐models of log‐ratio of ectomycorrhizal genera in bags relative to roots.
**Table S4** Model output from lme‐models of log‐ratio of ectomycorrhizal genera in soil‐filled relative to sand‐filled ingrowth meshbags.
**Table S5** Model output from PERMANOVA testing the effect of different soil substrates on ectomycorrhizal fungal community composition in ingrowth mesh bags.
**Table S6** Relative abundance of ectomycorrhizal fungi in ingrowth bags filled with different organic substrates.
**Table S7** Model output from models testing the effect of substrates in ingrowth meshbags on ectomycorrhizal fungal genera.
**Table S8** Output from post hoc Tukey HSD test on ectomycorrhizal genera that displayed preference towards any soil substrate.
**Table S9** Model output from PERMANOVA testing the effect of different sand substrates on ectomycorrhizal fungal community composition in ingrowth mesh bags.
**Table S10** Relative abundance of ectomycorrhizal fungi in ingrowth bags filled with sand.
Please note: Wiley is not responsible for the content or functionality of any Supporting Information supplied by the authors. Any queries (other than missing material) should be directed to the *New Phytologist* Central Office.

## Data Availability

Data and code needed to reproduce analyses are available on Dryad Digital Repository (doi: 10.5061/dryad.08kprr55q; Jörgensen *et al*., 2022). Sequence data are published in NCBI‐SRA under project PRJNA796466.
