# Supplementary material for: Do ectomycorrhizal exploration types reflect mycelial foraging strategies?
Source: New Phytol. 2022 Nov 23;237(2):576–84. doi: 10.1111/nph.18566 (PMC10098516; doi:10.1111/nph.18566)
Supplement: Supplementary file 1 — Fig. S1 Illustration of cafeteria setup. Table S1 Model output from lme‐models of the log‐ratio of ectomycorrhizal exploration types in bags relative to roots, and in soil bags relative to sand bags. Table S2 Relative abundance of ectomycorrhizal fungi on roots, and sand‐ or soil‐filled ingrowth bags. Table S3 Model output from lme‐models of log‐ratio of ectomycorrhizal genera in bags relative to roots. Table S4 Model output from lme‐models of log‐ratio of ectomycorrhizal genera in soil‐filled relative to sand‐filled ingrowth meshbags. Table S5 Model output from PERMANOVA testing the effect of different soil substrates on ectomycorrhizal fungal community composition in ingrowth mesh bags. Table S6 Relative abundance of ectomycorrhizal fungi in ingrowth bags filled with different organic substrates. Table S7 Model output from models testing the effect of substrates in ingrowth meshbags on ectomycorrhizal fungal genera. Table S8 Output from post hoc Tukey HSD test on ectomycorrhizal genera that displayed preference towards any soil substrate. Table S9 Model output from PERMANOVA testing the effect of different sand substrates on ectomycorrhizal fungal community composition in ingrowth mesh bags. Table S10 Relative abundance of ectomycorrhizal fungi in ingrowth bags filled with sand. Please note: Wiley is not responsible for the content or functionality of any Supporting Information supplied by the authors. Any queries (other than missing material) should be directed to the New Phytologist Central Office. [file NPH-237-576-s001.pdf]

***New Phytologist* Supporting Information**

Article title: Do ectomycorrhizal exploration types reflect mycelial foraging strategies?
Authors: Karolina Jörgensen, Karina E. Clemmensen, Håkan Wallander, Björn D. Lindahl
Article acceptance date: 1/10/2022

The following Supporting Information is available for this article:

Figure S1 Illustration of cafeteria setup

Table S1 Model output from lme-models of the log-ratio of ectomycorrhizal exploration types in bags relative to roots, and in soil bags relative to sand bags

Table S2 Relative abundance of ectomycorrhizal fungi on roots, and sand- or soil-filled ingrowth bags

Table S3 Model output from lme-models of log-ratio of ectomycorrhizal genera in bags relative to roots roots

Table S4 Model output from lme-models of log-ratio of ectomycorrhizal genera in soil-filled relative to sand-filled ingrowth meshbags

Table S5 Model output from PERMANOVA testing the effect of different soil substrates on ectomycorrhizal fungal community composition in ingrowth mesh bags

Table S6 Relative abundance of ectomycorrhizal fungi in ingrowth bags filled with different organic substrates

Table S7 Model output from models testing the effect of substrates in ingrowth meshbags on ectomycorrhizal fungal genera

Table S8 Output from post-hoc Tukey HSD test on ectomycorrhizal genera that displayed preference towards any soil substrate.

Table S9 Model output from PERMANOVA testing the effect of different sand substrates on ectomycorrhizal fungal community composition in ingrowth mesh bags

Table S10 Relative abundance of ectomycorrhizal fungi in ingrowth bags filled with sand

Figure S1. Illustration of cafeteria setup. Light gray point represents the location of the core that was used to extract roots, and analyse root-communities while the dark gray points represents the location of the ingrowth bags. The distance between the root core and the ingrowth bags was 0.5 m. All six substrate types (organic topsoil – low N deposition; organic topsoil – high N deposition; organic topsoil – P fertilised; mull soil; sand; sand with apatite) were included in all cafeterias and the order by which the ingrowth bags were placed in the cafeterias was randomised. In all sites (N=10), five replicate cafeterias were incubated.

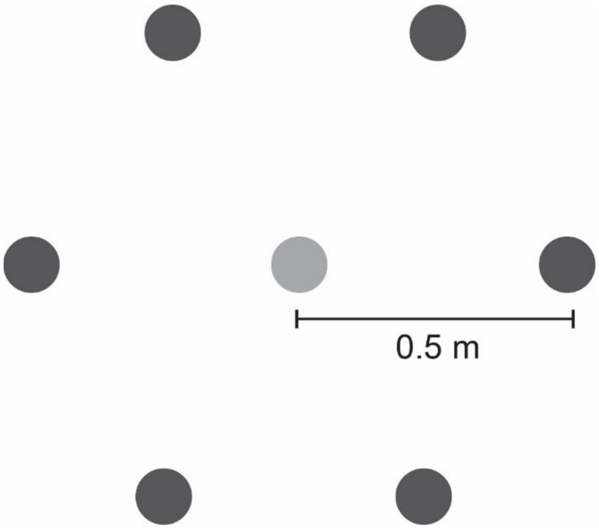

Table S1. Model output from mixed effects linear models testing the log-transformed ratio of the relative abundance of ectomycorrhizal fungal exploration types in ingrowth meshbags relative to roots and in soil-filled bags relative to sand-filled bags. Models were fitted with the log-ratio of relative abundances of ectomycorrhizal exploration types in ingrowth bags relative to on roots, or soil-filled bags relative to sand-filled bags as response variable, and exploration type as explanatory factor and cafeteria ID nested within site as a random variable.

	Sum of squares	Mean Sq	DF	Den DF	F value	Pr(>F)
Bags *vs.* roots	7.007	2.336	3	7.94	0.458	0.719
Soil bags *vs.* sand bags	27.182	9.061	3	8.03	1.374	0.319

Table S2. Overall relative abundance ((root mean + bag mean)/2) and mean relative abundance of ectomycorrhizal fungal genera on roots and in ingrowth meshbags, and in soil- or sand filled bags, respectively, mean relative abundance with standard error in parentheses. Mean values are based on all cafeterias.

	Overall	Roots	Bags	Sand bags	Soil bags
Amanita	0.030	0.006 (0.003)	0.054 (0.010)	0.016 (0.009)	0.072 (0.014)
Amphinema	0.153	0.033 (0.008)	0.272 (0.017)	0.401 (0.035)	0.212 (0.018)
Cenococcum	0.120	0.161 (0.032)	0.079 (0.010)	0.112 (0.022)	0.063 (0.010)
Cortinarius	0.061	0.108 (0.027)	0.015 (0.004)	0.02 (0.006)	0.012 (0.004)
Hyaloscypha	0.043	0.08 (0.019)	0.007 (0.004)	0.013 (0.011)	0.004 (0.002)
Hygrophorus	0.010	0.019 (0.007)	0 (0)	0 (0)	0 (0)
Lactarius	0.026	0.025 (0.009)	0.026 (0.007)	0.002 (0.001)	0.038 (0.010)
Piloderma	0.110	0.182 (0.031)	0.037 (0.006)	0.015 (0.004)	0.046 (0.009)
Pseudotomentella	0.025	0.017 (0.011)	0.033 (0.006)	0.056 (0.015)	0.022 (0.005)
Russula	0.098	0.118 (0.026)	0.077 (0.010)	0.009 (0.002)	0.109 (0.015)
Tomentella (incl. Thelephora)	0.122	0.097 (0.025)	0.146 (0.011)	0.12 (0.017)	0.158 (0.014)
Tylospora	0.072	0.019 (0.006)	0.124 (0.015)	0.135 (0.026)	0.119 (0.018)

Table S3. Model output from mixed effects linear models testing the log-transformed ratio of the relative abundance of ectomycorrhizal fungal genera in ingrowth meshbags relative to roots. Models were fitted with the log-ratio of the relative abundance of each genus (separately) in ingrowth bags relative to roots as a response variable and cafeteria ID nested within site as a random factor. * denote significant effects after correcting for multiple testing using the Benjamini-Hochberg method.

Genus	Estimate	Std. Error	df	t value	Pr(>|t|)	
Amanita	1.22	0.59	10	2.06	0.067	
Amphinema	2.56	0.32	30	7.98	<0.001	*
Cenococcum	-1.04	0.57	9	-1.82	0.101	
Cortinarius	-2.53	0.56	9	-4.49	0.002	*
Hyaloscypha	-3.99	0.55	9	-7.32	<0.001	*
Hygrophorus	-5.08	0.43	5	-11.68	<0.001	*
Lactarius	-0.66	0.71	6	-0.94	0.385	
Piloderma	-2.23	0.43	7	-5.24	0.001	*
Pseudotomentella	-1.26	0.66	6	-1.92	0.106	
Russula	-0.82	0.46	9	-1.79	0.105	
Tomentella	0.88	0.31	33	2.81	0.008	*
Tylospora	1.31	0.42	7	3.12	0.018	*

Table S4. Model output from mixed effects linear models testing the log-transformed ratio of the relative abundance of ectomycorrhizal fungal genera in soil-filled bags relative to sand-filled ingrowth meshbags. Models were fitted with the log-ratio of the relative abundance of each genus (separately) in soil-filled bags relative to sand-filled bags as a response variable and cafeteria ID nested within site as a random factor. * denote significant effects after correcting for multiple testing using the Benjamini-Hochberg method.

Genus	Estimate	Std. Error	df	t value	Pr(>|t|)	
Amanita	3.06	1.04	10	2.93	0.015	*
Amphinema	-0.63	0.23	30	-2.76	0.01	*
Cenococcum	-0.27	0.44	37	-0.62	0.54	
Cortinarius	0.03	0.49	34	0.06	0.954	
Hyaloscypha	-0.07	0.42	38	-0.17	0.869	
Hygrophorus	0.59	0.5	12	1.18	0.262	
Lactarius	3.37	0.72	21	4.69	<0.001	*
Piloderma	1.53	0.5	36	3.07	0.004	*
Pseudotomentella	0.14	0.67	8	0.2	0.845	
Russula	3.96	0.52	8	7.64	<0.001	*
Tomentella	0.44	0.26	8	1.67	0.132	
Tylospora	0.32	0.56	21	0.57	0.577	

Table S5. Model output from PERMANOVA with 1000 permutations testing the effect of different soil substrates in ingrowth mesh bags on ectomycorrhizal fungal community composition.

	Df	Sum of squares	R2	F	Pr(>F)
Soil substrate	3	2.301	0.060	6.454	<0.001
Site no.	9	7.914	0.206	7.400	<0.001
Cafeteria ID	37	12.684	0.330	2.885	<0.001
Residual	131	15.566	0.405		
Total	180	38.464	1.000		

Table S6. Mean relative abundance (based on all cafeterias) of ectomycorrhizal fungal genera in ingrowth meshbags containing different organic substrates, standard error in parentheses.

	Relative abundance			
	Organic Central Sweden	Organic Southern Sweden	P fertilised Organic Southern Sweden	Mull soil Central Sweden
Amanita	0.06 (0.025)	0.130 (0.037)	0.067 (0.028)	0.031 (0.015)
Amphinema	0.303 (0.043)	0.097 (0.023)	0.174 (0.029)	0.274 (0.040)
Cenococcum	0.019 (0.010)	0.107 (0.027)	0.079 (0.022)	0.047 (0.018)
Cortinarius	0.005 (0.002)	0.008 (0.003)	0.015 (0.009)	0.020 (0.015)
Hyaloscypha	0.009 (0.009)	0 (0)	0 (0)	0.006 (0.004)
Hygrophorus	0 (0)	0 (0)	0.001 (0.001)	0 (0)
Lactarius	0.034 (0.018)	0.058 (0.026)	0.057 (0.024)	0.003 (0.001)
Piloderma	0.089 (0.028)	0.041 (0.013)	0.023 (0.009)	0.033 (0.014)
Pseudotomentella	0.021 (0.011)	0.016 (0.008)	0.029 (0.012)	0.022 (0.007)
Russula	0.06 (0.016)	0.142 (0.033)	0.108 (0.030)	0.124 (0.035)
Tomentella (incl. Thelephora)	0.213 (0.031)	0.086 (0.019)	0.179 (0.030)	0.155 (0.026)
Tylospora	0.123 (0.037)	0.086 (0.030)	0.141 (0.041)	0.126 (0.036)

Table S7. Model output from ANOVA of mixed effects linear models testing differences in square-root transformed relative abundance of ectomycorrhizal fungal genera in different organic substrates. * denote significant effects after correcting for multiple testing using the Benjamini-Hochberg method.

	Sum of Sq	Mean Sq	DF	Den DF	F value	Pr(>F)	
Amanita	0.206	0.069	3	30	0.96	0.423	
Amphinema	1.565	0.522	3	85.7	14.29	<0.001	*
Cenococcum	0.601	0.2	3	103.9	6.73	<0.001	*
Cortinarius	0.008	0.003	3	94.6	0.24	0.865	
Hyaloscypha	0.021	0.007	3	110.6	2.14	0.099	
Hygrophorus	0.003	0.001	3	48	1.19	0.322	
Lactarius	0.204	0.068	3	61.2	1.99	0.125	
Piloderma	0.172	0.057	3	105.6	1.98	0.121	
Pseudotomentella	0.013	0.004	3	50.3	0.28	0.836	
Russula	0.136	0.045	3	100.1	1.44	0.235	
Tomentella	0.996	0.332	3	93.9	9.7	<0.001	*
Tylospora	0.008	0.003	3	60.1	0.07	0.978	

Table S8. Model output from post-hoc Tukey HSD test of the square-root transformed relative abundance of tree ectomycorrhizal genera that showed preference towards any of the soil substrates in ingrowth mesh bags.

	Estimate	SE	df	t ratio	p-value
Amphinema					
F-M	0.098	0.050	85.8	1.96	0.212
F-P	0.196	0.050	86.1	3.86	0.001
F-RH	0.309	0.050	85.5	6.23	<0.001
M-P	0.097	0.050	85.8	1.94	0.218
M-RH	0.211	0.049	85.3	4.30	<0.001
P-RH	0.114	0.050	85.5	2.29	0.108
Cenococcum					
F-M	-0.064	0.040	106	-1.58	0.393
F-P	-0.149	0.041	107	-3.68	0.002
F-RH	-0.155	0.041	107	-3.84	0.001
M-P	-0.085	0.041	107	-2.11	0.158
M-RH	-0.092	0.041	107	-2.27	0.113
P-RH	-0.006	0.041	107	-0.16	0.999
Tomentella					
F-M	0.134	0.046	94.5	2.91	0.023
F-P	0.047	0.047	95.0	1.01	0.743
F-RH	0.230	0.046	94.5	4.99	<0.001
M-P	-0.087	0.046	94.5	-1.89	0.240
M-RH	0.096	0.046	94.0	2.10	0.162
P-RH	0.183	0.046	94.5	3.97	<0.001

Table S9. Model output from PERMANOVA with 1000 permutations testing the effect of different sand substrates in ingrowth mesh bags on ectomycorrhizal fungal community composition.

	Df	Sum of squares	R2	F	Pr(>F)
Sand substrate	1	0.083	0.006	1.045	0.473
Site no.	9	4.330	0.328	6.046	0.694
Cafeteria ID	38	6.071	0.460	2.008	0.679
Residual	34	2.710	0.205		
Total	82	13.190	1.000		

Table S10. Mean relative abundance of ectomycorrhizal genera in sand or apatite filled ingrowth meshbags, standard error in parentheses. Relative abundances are based on all cafeterias.

	Sand	Apatite
Amanita	0.015 (0.009)	0.017 (0.015)
Amphinema	0.424 (0.049)	0.377 (0.049)
Cenococcum	0.113 (0.030)	0.110 (0.032)
Cortinarius	0.025 (0.009)	0.015 (0.008)
Hyaloscypha	0.002 (0.001)	0.025 (0.023)
Hygrophorus	0 (0)	0 (0)
Lactarius	0.004 (0.002)	0 (0)
Piloderma	0.014 (0.005)	0.017 (0.007)
Pseudotomentella	0.054 (0.016)	0.058 (0.025)
Russula	0.011 (0.004)	0.007 (0.003)
Tomentella (incl. Thelephora)	0.111 (0.026)	0.129 (0.021)
Tylospora	0.140 (0.036)	0.129 (0.037)
